# Health literacy of patients on oral anticoagulation treatment- individual and social determinants and effect on health and treatment outcomes

**DOI:** 10.1186/s12889-021-11259-w

**Published:** 2021-07-09

**Authors:** Ana Cristina Cabellos-García, Antonio Martínez-Sabater, Miguel Ángel Díaz-Herrera, Vicente Gea-Caballero, Enrique Castro-Sánchez

**Affiliations:** 1 Hospital Universitario y politécnico La Fe, Valencia, Spain; 2 Health Research Institut La Fe, Research Group GREIACC, Valencia, Spain; 3 Nursing Department, Universitat de València, Valencia, Spain; 4 Facultat d’Infermeria i Podologia. Nursing Care and Education Research Group. (GRIECE). Grupo Investigación en Cuidados (INCLIVA), Hospital Clínico Universitario, Valencia, Spain; 5 Direcció d’Atenció Primaria Costa Ponent, Institut Català de la Salut, Avinguda de la Gran via de l’Hospitalet, 199-203, 08908 L’Hospitalet de Llobregat, Barcelona, Spain; 6 Unitat de Suport a la Recerca Costa de Ponent, Institut Universitari d’Investigació en Atenció Primària Jordi Gol (IDIAP Jordi Gol), 08940 Cornellà de Llobregat, Spain; 7 Escuela de Enfermería La Fe, centre affiliated to Universitat de Valencia, Valencia, Spain; 8 School of Health Sciences, University of London, London, UK; 9 National Institute for Health Research Health Protection Research Unit in Healthcare-Associated Infections and Antimicrobial Resistance at Imperial College London, London, UK

**Keywords:** Health literacy, Anticoagulants, Acenocoumarol, Treatment adherence and compliance, Self-management, Drug-related side effects and adverse reaction, Health services

## Abstract

**Background:**

Assessment health literacy in people with cardiovascular health problems would facilitate the development of appropriate health strategies for the care and reduction of complications associated with oral anticoagulation therapy. Aim: To evaluate the relationship between health literacy and health and treatment outcomes (concordance with oral anticoagulants, Normalized Ratio control and occurrence of complications) in patients with cardiovascular pathology.

**Methods:**

Observational, analytic and cross-sectional study carried out on 252 patients with cardiovascular pathology (atrial fibrillation, flutter or valve prosthesis), aged 50–85 years, accessing primary care services in Valencia (Spain) in 2018–2019. Variables referring to anticoagulant treatment with vitamin K antagonists (years of treatment, adequate control, polypharmacy and occurrence of complications, among others) and health literacy (Health Literacy Questionnaire) were analysed.

**Results:**

All dimensions of health literacy were significantly related to the level of education (*p* < 0.02), social class (*p* < 0.02), an adequate control of acenocoumarol (*p* < 0.001), frequentation of health services (p < 0.001), information by patients to health professionals about anticoagulant treatment (*p* < 0.03), emergency care visits (*p* < 0.001) and unscheduled hospital admissions (*p* < 0.001).

**Conclusion:**

Health literacy has a relevant influence on the adequate self-management of anticoagulation treatment and the frequency of complications. The different dimensions that comprise health literacy play an important role, but the “social health support” dimension seems to be essential for such optimal self-management.

**Trial registration:**

ACC-ACE-2016-01. Registration date: December 2015.

## Background

Cardiovascular diseases are the leading cause of death worldwide. Atrial fibrillation (AF) is the most frequent arrhythmia, associated with high mortality and morbidity [1], and with an estimated prevalence in Spain of over 4.4%. Therapeutic management of AF requires modification of cardiovascular risk factors and use of drugs [1, 2] such as oral anticoagulants (OAC). Among these, both vitamin K antagonists (VKAs) and direct oral anticoagulants (DOACs) are effective in preventing embolic events [2]. Because the anticoagulant effect of DOACs is difficult to quantify, continuous monitoring and education are necessary to ensure therapeutic follow-up, reflecting the complexity of DOACs use [3, 4].

The recommendations for switching from VKAs to DOACs due to the suboptimal control of the international normalized ratio (INR) were eased in Spain in 2012. At present, however, the legal requirement for a visa and the high financial cost of DOACs prescribing make it difficult to obtain widespread access for patients [5]. For such reasons, VKAs continue to be the most frequently used OAC in Spain, despite the need for routine blood tests and an exquisite clinical control [6]. Further, it is vital that patients demonstrate adequate self-care, along with treatment concordance and sufficient knowledge to avoid or mitigate adverse effects, to maintain the effectiveness of this treatment.

With these requirements in place, health literacy (HL) could be an essential factor for the self-management of anticoagulation treatment, in line with other cardiovascular diseases [7]. Conceptually, HL encompasses different aspects such as empowerment, as well as competencies to understand, evaluate and use health information to make decisions, including the use of health services [8]. Some preliminary studies exploring the effect of HL on awareness about AF and medication concordance have demonstrated that people with inadequate or insufficient levels of HL have poorer health outcomes, have less knowledge about their health problems and diseases, use preventive services less, and suffer from higher mortality [9, 10].

So far, HL has been linked to the health conditions and outcomes of people with cardiovascular disease. Therefore, we believe that effective self-care, adherence to treatment with oral anticoagulants and prevention of complications could be directly related to the level of health literacy.

However, the existing literature has directly associated higher levels of HL with greater knowledge of the disease, but the association among HL, adherence to anticoagulant therapy and the occurrence of adverse events has presented highly variable results and studies have been scarce [11].

Because of this, we propose identifying the level of HL in the local population on anticoagulation treatment and exploring the relationship between HL and several health and clinical outcomes. In addition, our work will provide information about the relationship between the different dimensions that make up HL, including the social dimension as a novel aspect, and compliance with OAC treatment, INR control and the occurrence of complications.

## Methods

### Study design

Observational, analytic and cross-sectional study.

### Population

We focused on 6 areas within the Xàtiva/Ontinyent Health Department (Valencia, Spain), which were randomly selected from the 17 areas of the department (chose 6 numbers at random, choosing the areas that corresponded with the number extracted). During the study period (January 1, 2018 to April 30, 2019), all patients who came to the nursing consultation were invited to fill out the HL questionnaire. For a population of *N* = 730 patients (data provided by the health department’s pharmacy service), the smallest representative sample we calculated is 252 patients (confidence level 95%, margin of error 5%, population proportion 50%). Recruitment ended after reaching the minimum representative study sample (because the questionnaire was long, with complex questions and patients in many cases refused to stay and wait 15–20 min more in the health centre).

The eligibility criteria were: Patients who are between 50 and 85 years old, have cardiovascular disease, mainly arrhythmias (atrial fibrillation or flutter) or valvular disease, and have received VKAs treatment for at least 6 months. Exclusion criteria included vision or hearing impairment that prevented the completion of the HL questionnaire, illiteracy, discontinued treatment with VKAs, severe neurocognitive or mental health problems that prevented the patient from understanding their pathological conditions, and finally patients whose treatment with VKAs was administered by another person. Methods of selection of participants were to offer participation in the study to all patients who came to the nursing consultation (both on a scheduled and on demand) and met the selection criteria.

The study was conducted in rural areas with a population of 2300 to 8000 inhabitants in each primary health care center, where the distance to the health care center is usually short and can be reached on foot by most citizens.

### Study assessment parameters

Data were obtained on the following variables:

- Socio-demographic: Age, sex, educational level (without studies/basic education/university education) and self-perceived social class (low/medium/high).
- Clinical: Main diagnosis, obesity (BMI > 30), high blood pressure (HBP), polypharmacy (prescription ≥5 drugs), tobacco use (non-smoker/smoker) and occurrence of complications (emergency care or unscheduled hospital admissions during the previous six months).
- VKA treatment: Years of treatment, reporting of VKA treatment by patients to other health professionals, number of controls in 6 months, control of VKA treatment [good control if ~ 65% INR measurements within range, with measurement by direct method, for at least 6 months] [1, 2]
- HL: The Health Literacy Questionnaire (HLQ) [12] was used to assess the level of HL. This questionnaire assesses 9 different dimensions, consists of 44 items and has been validated for Spanish speakers.
- Dimension 1 (D1): Feeling understood and supported by health care providers.
- Dimension 2 (D2): Having enough information to manage my health.
- Dimension 3 (D3) Actively managing my health.
- Dimension 4 (D4) Social health support.
- Dimension 5 (D5) Assessment of health information.
- Dimension 6 (D6) Ability to actively participate with health care providers.
- Dimension 7 (D7) Navigation through the health system.
- Dimension 8 (D8) Ability to find good health information.
- Dimension 9 (D9) Understanding health information well enough to know what to do.

The scores for dimensions 1 to 5 are set to four values ​​(completely disagree/disagree/agree/strongly agree), and the scores for dimensions 6 to 9 are set to 5 values ​​(cannot be done or always have difficulties/usually difficult/ Sometimes difficult/ usually have ease/ always have ease). According to the author’s request, an independent score was established for each dimension [13].

Sociodemographic variables were collected by means of a patient interview, while clinical variables and those referring to anticoagulant treatment with VKA were collected from the medical records and prescriptions. With regard to the health literacy variable, the questionnaire was administered to patients by means of a direct interview with the principal investigator.

### Statistical analysis

To analyse quantitative variables were used central tendency and dispersion measures. Absolute and relative frequencies, expressed in percentages, were used for qualitative variables. Parametric and non-parametric tests were performed to evaluate the relationship between different variables and dimensions of HLQ. The statistical significance level was determined to be *p* < 0.05. Were analysed all data using SPSS Statistics for Windows (version 23, Spanish, Armonk, NY, IBM).

### Ethical considerations

The study was approved by the Ethics and Clinical Research Committee of the Primary Care Region of Valencia (Ref.ACC-ACE-2016-01). All participants have provided their written consent to participate in the study and the data of the patients included in the study have been anonymized at all times.

## Results

### Description of the sample

The response rate was 35% (252 patients responded to the questionnaire). The average age was 74.3 +/− 7.3 years, with ~ 90% over 65-years old. Forty-two percent were women, and 74.9% had AF as primary diagnosis. With respect to their educational level, 50% of participants had basic studies, while 40.1% did not. Of the 252 participants, 49.2% had complications and 11.9% required hospital admission (Table 1).

**Table 1:** Characteristics of participants (*n* = 252)

		M/n	Sd/ %
Age		74.38	7.35
	< 65 years	26	10.3%
	= > 65 years	226	89.7%
Sex	Woman	107	42.5%
	Man	145	57.5%
Education Level	Without Studies	101	40.1%
	Basic Education	126	50.0%
	Higher Education	25	9.9%
Social Class	Low	33	13.1%
	Middle	206	81.7%
	High	13	5.2%
Main Diagnosis	Atrial Fibrillation	188	74.9%
	Atrial Flutter	7	2.8%
	Aortic Prosthesis	33	13.1%
	Mitral Prosthesis	23	9.2%
Years of treatment		7.6	6.79
Good control of acenocoumarol	No	114	45,2%
	Yes	138	54,8%
Appearance of complications	No	128	50.8%
	Yes	124	49.2%
Emergency assistance last 6 months	No	129	51.2%
	Yes	123	48.8%
Hospital admission last 6 months	No	222	88.1%
	Yes	30	11.9%
Polypharmacy	No	86	34.1%
	Yes	166	65.9%

*M* Mean, *n* number of cases, *Sd* Standard deviation; %, percentage

### Relationships between HL dimensions and variables

The average score of the participants on each HL dimension was slightly higher than expected (average of the scale) in all dimensions (Table 2), but mainly in dimension D4. The relationship between the HL dimensions and the different study variables is presented in Tables 3 and 4.

**Table 2:** Average score of HL dimensions

Dimension	Mean	Standard error of the mean	Standard deviation	Median	Maximum	Minimum	25 Percentile	75 Percentile
Dimension 1	3.201	.027	.429	3.125	4.000	1.500	3.000	3.500
Dimension 2	2.657	.033	.517	2.750	4.000	1.000	2.250	3.000
Dimension 3	2.817	.032	.511	2.800	4.000	1.000	2.600	3.000
Dimension 4	3.494	.028	.437	3.600	4.000	1.400	3.200	3.800
Dimension 5	2.387	.043	.686	2.400	4.000	1.000	2.000	2.800
Dimension 6	4.106	.038	.596	4.200	5.000	1.200	3.800	4.600
Dimension 7	3.507	.041	.654	3.667	5.000	1.167	3.083	4.000
Dimension 8	3.052	.054	.851	3.200	5.000	1.000	2.400	3.800
Dimension 9	3.268	.055	.871	3.400	5.000	1.400	2.600	4.000

**Table 3:** Relationship and statistical significance between variables and HL dimensions

		D1	D2	D3	D4	D5	D6	D7	D8	D9
**Age** ^D^	***Sig.***	.022	−.105	−.104	−.030	−.223**	−.118	−.189*	−.282**	−.261**
< 65 years	*Mean (SD)*	3.18 (0.4)	2.73 (0.47)	2.86 (0.58)	3.48 (0.33)	2.59 (0.78)	4.35 (0.49)	3.74 (0.58)	3.42 (0.76)	3.59 (0.85)
	Median (IQR)	3.13 (0.50)	2.75 (0.50)	3.00 (0.80)	3.40 (0.60)	2.60 (1.20)	4.40 (1.00)	3.83 (0.67)	3.50 (1.20)	3.80 (1.60)
= > 65 years	*Mean (SD)*	3.2 (0.43)	2.65 (0.52)	2.81 (0.5)	3.5 (0.45)	2.36 (0.67)	4.08 (0.6)	3.48 (0.66)	3.01 (0.85)	3.23 (0.87)
	Median (IQR)	3.13 (0.50)	2.75 (0.75)	2.80 (0.40)	3.60 (0.60)	2.40 (0.80)	4.20 (0.60)	3.50 (1.00)	3.00 (1.20)	3.40 (1.60)
**Sex** ^A^	***Sig.***	.496	.055	.298	.137	.096	.217	.142	028*	.015*
Woman	*Mean (SD)*	3.18 (0.46)	2.58 (0.54)	2.78 (0.53)	3.44 (0.5)	2.3 (0.7)	4.05 (0.64)	3.44 (0.66)	2.92 (0.84)	3.11 (0.85)
	Median (IQR)	3.00 (0.50)	2.50 (0.75)	2.80 (0.40)	3.60 (0.60)	2.40 (1.00)	4.00 (1.00)	3.50 (1.00)	3.00 (1.40)	3.20 (1.40)
Man	*Mean (SD)*	3.22 (0.4)	2.71 (0.5)	2.85 (0.5)	3.53 (0.38)	2.45 (0.67)	4.15 (0.56)	3.56 (0.65)	3.15 (0.85)	3.38 (0.87)
	Median (IQR)	3.25 (0.50)	2.75 (0.75)	2.80 (0.60)	3.60 (0.40)	2.40 (0.80)	4.20 (0.80)	3.67 (0.83)	3.20 (1.20)	3.40 (1.40)
**Education Level** ^B,C^	***Sig.***	< .001**	< .001**	< .001**	.016*	< .001**	< .001**	< .001**	< .001**	< .001**
Without Studies	*Mean (SD)*	3.06 (0.4)	2.33 (0.43)	2.51 (0.45)	3.42 (0.41)	1.94 (0.54)	3.85 (0.60)	3.08 (0.54)	2.38 (0.57)	2.56 (0.58)
	Median (IQR)	3.00 (0.25)	2.25 (0.5)	2.60 (0.60)	3.60 (0.60)	2.00 (0.80)	4.00 (0.60)	3.17 (0.83)	2.40 (0.80)	2.40 (0.80)
Basic Education	*Mean (SD)*	3.28 (0.44)	2.83 (0.47)	2.96 (0.42)	3.52 (0.47)	2.58 (0.56)	4.23 (0.53)	3.72 (0.57)	3.39 (0.66)	3.62 (0.67)
	Median (IQR)	3.25 (0.50)	3.00 (0.5)	3.00 (0.40)	3.80 (0.40)	2.60 (0.60)	4.20 (0.60)	3.83 (0.50)	3.60 (1.00)	3.80 (0.60)
Higher Education	*Mean (SD)*	3.4 (0.36)	3.11 (0.32)	3.36 (0.39)	3.67 (0.26)	3.26 (0.56)	4.54 (0.49)	4.16 (0.42)	4.06 (0.63)	4.35 (0.49)
	Median (IQR)	3.25 (0.50)	3.00 (0.25)	3.20 (0.80)	3.80 (0.20)	3.20 (1.00)	4.60 (0.60)	4.00 (0.33)	4.00 (0.80)	4.40 (0.60)
**Social Class** ^**C**^	***Sig.***	.019*	< .001**	< .001**	.003*	< .001**	< .001**	< .001**	< .001**	< .001**
Low	*Mean (SD)*	2.98 (0.44)	2.3 (0.39)	2.41 (0.53)	3.38 (0.39)	1.81 (0.55)	3.76 (0.56)	3.04 (0.58)	2.25 (0.64)	2.43 (0.61)
	Median (IQR)	3.00 (0.50)	2.25 (0.50)	2.4 (0.80)	3.40 (0.40)	1.80 (0.80)	3.80 (0.80)	3.00 (0.67)	2.20 (0.40)	2.20 (0.80)
Middle	*Mean (SD)*	3.23 (0.42)	2.71 (0.51)	2.87 (0.49)	3.5 (0.45)	2.45 (0.66)	4.15 (0.59)	3.56 (0.65)	3.14 (0.82)	3.35 (0.83)
	Median (IQR)	3.25 (0.50)	2.75 (0.75)	3.00 (0.60)	3.60 (0.60)	2.60 (0.80)	4.20 (0.80)	3.67 (0.83)	3.20 (1.20)	3.40 (1.40)
High	*Mean (SD)*	3.27 (0.33	2.77 (0.5)	3 (0.34)	3.72 (0.29)	2.88 (0.67)	4.34 (0.51)	3.91 (0.31)	3.66 (0.64)	4.10 (0.56)
	Median (IQR)	3.25 (0.50)	2.75 (0.25)	3.00 (0.20)	3.80 (0.0)	3.00 (0.40)	4.20 (0.60)	4.00 (0.17)	4.00 (0.80)	4.20 (0.20)
**Polypharmacy** ^**A**^	***Sig.***	.255	< .001**	.005*	.780	< .001**	.367	.004*	< .001**	< .001**
No	*Mean (SD)*	3.24 (0.4)	2.82 (0.46)	2.94 (0.58)	3.48 (0.4)	2.62 (0.68)	4.15 (0.63)	3.67 (0.64)	3.4 (0.83)	3.58 (0.84)
	Median (IQR)	3.25 (0.50)	3.00 (0.50)	3.00 (0.40)	3.60 (0.60)	2.60 (0.60)	4.20 (0.80)	3.83 (0.50)	3.60 (1.20)	3.80 (1.00)
Yes	*Mean (SD)*	3.18 (0.44)	2.57 (0.53)	2.75 (0.46)	3.5 (0.46)	2.26 (0.66)	4.08 (0.58)	3.42 (0.65)	2.87 (0.81)	3.10 (0.84)
	Median (IQR)	3.00 (0.50)	2.50 (0.75)	2.80 (0.40)	3.60 (0.60)	2.40 (1.00)	4.10 (0.60)	3.33 (0.83)	2.80 (1.40)	3.10 (1.40)
**Obesity** ^**A**^	***Sig.***	.895	.288	.113	.525	.030*	.318	.142	.030*	.018*
No	*Mean (SD)*	3.2 (0.43)	2.69 (0.52)	2.86 (0.51)	3.48 (0.45)	2.46 (0.7)	4.14 (0.55)	3.56 (0.65)	3.15 (0.84)	3.38 (0.86)
	Median (IQR)	3.13 (0.50)	2.75 (0.75)	2.80 (0.60)	3.60 (0.60)	2.60 (1.00)	4.20 (0.80)	3.67 (0.83)	3.20 (1.30)	3.40 (1.30)
Yes	*Mean (SD)*	3.2 (0.42)	2.62 (0.52)	2.76 (0.51)	3.52 (0.41)	2.28 (0.65)	4.06 (0.66)	3.43 (0.66)	2.91 (0.86)	3.11 (0.86)
	Median (IQR)	3.13 (0.50)	2.50 (0.75)	2.80 (0.50)	3.60 (0.50)	2.40 (1.00)	4.20 (0.60)	3.50 (0.83)	3.00 (1.50)	3.20 (1.50)

* *p* < .05; ** *p* < .001; A, T-Student tests; B, Anova; C, Kruskal-Wallis; D,Mann-Whitney U; SD, Standar desviation; IQR, Interquartile range

**Table 4:** Statistical relationship between variables related to the treatment of OACs and occurrence of complications with HL dimensions

		D1	D2	D3	D4	D5	D6	D7	D8	D9
Good VKAs control ^**A**^	***Sig.***	< .001**	< .001**	< .001**	.006*	< .001**	< .001**	< .001**	< .001**	< .001**
No	*Mean (SD)*	3.06 (0.41)	2.37 (0.42)	2.54 (0.50)	3.41 (0.46)	2.02 (0.58)	3.86 (0.60)	3.13 (0.61)	2.45 (0.64)	2.64 (0.67)
Yes	*Mean (SD)*	3.31 (0.41)	2.89 (0.46)	3.04 (0.39)	3.56 (0.40)	2.68 (0.62)	4.30 (0.50)	3.81 (0.52)	3.54 (0.67)	3.77 (0.65)
**Adequate frequency** ^**A**^	***Sig.***	< .001**	< .001**	< .001**	.001*	< .001**	< .001**	< .001**	< .001**	< .001**
No	*Mean (SD)*	3.06 (0.37)	2.36 (0.40)	2.50 (0.48)	3.37 (0.45)	1.97 (0.60)	3.86 (0.60)	3.12 (0.61)	2.46 (0.66)	2.66 (0.71)
Yes	*Mean (SD)*	3.28 (0.44)	2.83 (0.50)	3.00 (0.42)	3.56 (0.41)	2.63 (0.60)	4.25 (0.54)	3.74 (0.56)	3.41 (0.74)	3.63 (0.74)
**Inform that he takes VKAs** ^**D**^	***Sig.***	.027*	.004*	< .001**	.014*	.005*	.001*	.005*	< .001**	< .001**
No	*Mean (SD)*	3.00 (0.46)	2.32 (0.56)	2.28 (0.53)	3.30 (0.37)	1.99 (0.68)	3.71 (0.70)	3.16 (0.55)	2.33 (0.74)	2.60 (0.88)
Yes	*Mean (SD)*	3.21 (0.41)	2.68 (0.49)	2.86 (0.46)	3.51 (0.43)	2.42 (0.67)	4.15 (0.56)	3.54 (0.63)	3.12 (0.82)	3.40 (0.83)
**Complications** ^**A**^	***Sig.***	< .001**	< .001**	< .001**	.002*	< .001**	< .001**	< .001**	< .001**	< .001**
No	*Mean (SD)*	3.31 (0.40)	2.91 (0.44)	3.05 (0.40)	3.57 (0.37)	2.74 (0.59)	4.31 (0.51)	3.85 (0.54)	3.60 (0.66)	3.82 (0.63)
Yes	*Mean (SD)*	3.08 (0.41)	2.38 (0.44)	2.57 (0.49)	3.40 (0.48)	2.02 (0.57)	3.88 (0.59)	3.14 (0.55)	2.48 (0.61)	2.69 (0.68)
**Emergency assistance** ^**A**^	***Sig.***	< .001**	< .001**	< .001**	.002*	< .001**	< .001**	< .001**	< .001**	< .001**
No	*Mean (SD)*	3.31 (0.40)	2.91 (0.43)	3.04 (0.40)	3.57 (0.37)	2.74 (0.58)	4.31 (0.51)	3.85 (0.54)	3.60 (0.66)	3.83 (0.63)
Yes	*Mean (SD)*	3.08 (0.42)	2.38 (0.44)	2.57 (0.49)	3.40 (0.48)	2.01 (0.57)	3.88 (0.60)	3.13 (0.54)	2.47 (0.60)	2.67 (0.67)
**Hospital admission** ^**A**^	***Sig.***	.001*	< .001**	< .001**	.073	< .001**	< .001**	< .001**	< .001**	< .001**
No	*Mean (SD)*	3.23 (0.40)	2.70 (0.50)	2.86 (0.48)	3.51 (0.42)	2.44 (0.67)	4.16 (0.55)	3.57 (0.63)	3.15 (0.82)	3.36 (0.83)
Yes	*Mean (SD)*	2.95 (0.54)	2.31 (0.48)	2.43 (0.54)	3.36 (0.47)	1.92 (0.58)	3.68 (0.70)	3.03 (0.57)	2.28 (0.64)	2.52 (0.77)

* *p* < .05; ** *p* < .001; A, T-Student tests; B, Anova; C, Kruskal-Wallis; D, Mann-Whitney U

The ANOVA calculations of the different HL dimensions according to the **level of studies** were statistically significant, indicating differences in scores between two or more of the study level groups. The results showed that D2, D3, D5, D6, D7, D8 and D9 had differences between the three study level groups. The “without studies” group obtained the lowest mean scores on all dimensions, in contrast to the “university education” group with higher means.

For **social class**, all Kruskal-Wallis tests of the different HL dimensions were statistically significant (*p* < .05) implying differences in scores between two or more social class groups. Dimensions D5, D8 and D9, were explained by social class at 13–17%, suggesting a large social class effect on these dimensions. D5 and D9 showed differences between all social class groups, with lower median scores for lower-class than middle-class participants. In turn, the median scores in the middle class group were lower than those obtained by participants from the higher socioeconomic stratum.

For variables related to medication use, there were statistically significant differences for **“reports taking acenocoumarol”** in all HL dimensions and for **“polypharmacy”** in all dimensions except D1, D4 and D6 (Table 3). Non-polymedicated participants and those reporting treatment with VKA had higher mean scores on all dimensions.

Regarding the **control of acenocoumarol** and **frequency of controls**, statistically significant differences were obtained in all dimensions of the questionnaire. In all cases, participants with good anticoagulant control and adequate frequency obtained higher averages than those without good control or suboptimal control frequency. Additionally, the analysis indicated a small effect for the control of acenocoumarol and the frequency of controls on D4 scores. An average effect on the D1 scores for VKA control and on the D1 and D6 dimensions for proper control frequency is shown. Finally, there was a large effect on the scores of the other HL dimensions for drug control and control frequency (Table 4).

In relation to **cardiovascular disease risk factors**, tobacco use and HBP showed no statistically significant differences (*p* > .05) on any dimension. In contrast, statistically significant differences in the D5, D8 and D9 dimensions were observed in obese participants, with lower mean scores for these participants.

The occurrence of **complications** presented (*p* < .05) in the scores of all dimensions of the HL questionnaire. In all cases, the scores of patients without complications were higher (Table 4). The same was true for **emergency department attendance** in the previous 6 months. In both cases, a very large effect size was shown in all HL dimensions except D1, D4 and D6.

Finally, **hospital admissions** showed statistically significant differences (*p* < .05) in all dimensions for unscheduled hospitalizations in the previous 6 months, except in dimension D4. In all cases, the mean scores of patients with unscheduled hospital admissions were lower than in the rest of the patients (Table 4).

## Discussion

In our study in patients with cardiovascular pathology, health literacy actively influenced the adequate self-management of anticoagulation treatment, the appearance of complications and the unscheduled use of health services. Patients with higher scores on HL dimensions had better control of OAC treatment and optimal frequency of visits. They also reported confidence about their information management skills, empowerment, and self-efficacy, presenting better self-care. These results coincide with those demonstrated by some authors [14–17].

Therapeutic concordance with OACs was significantly related to the scores of all HL dimensions, with 54.8% of participants presenting an adequate control of OAC treatment. These results are similar to another study carried out in Valencia [18], where 53.9% presented good control, and slightly higher than the 47.5% of patients in Time in Therapeutic Range (TTR) in Granada [19]. Reading et al. [20] related inadequate HL to a lack of pharmacological adherence in AF, while another study associated a poor control of VKA (TTR < 50%) with limited HL, especially in patients over 65 [21]. However, other studies have reported equivocal relations between HL and treatment adherence; for example, HL as measured with the S-TOFHLA scale was associated with OAC treatment adherence but not with TTR [22, 23]. A 2017 study [24] using the SAHLPA scale linked inadequate level of HL to greater cognitive impairment and the need for help to take the treatment properly, but the multivariate analysis could not correlate an inadequate level of HL with having a TTR in the range. It is possible that these studies were unrelated because the scales were only used to evaluate recognition and ability to read certain words, not the different dimensions and skills that comprise HL. Because of this, patients may receive high scores in the scales yet lack more complex treatment-focused skills.

With respect to the occurrence of complications associated with OAC treatment, our study showed a significant association with scores in all HL dimensions, with higher-scoring patients experiencing fewer complications, lower emergency department attendance and hospitalization rates. Complications occurred in 49.2% of participants (228 patients), well above the 9% shown in a 2014 study [25]. This difference could be due to the origin of the information about the occurrence of complications, extracted from medical records in our case, while the 2014 study [25] used patient-reported complications which may have been affected by recall bias, particularly as the study noted that more than 50% of patients did not recognize emergency situations.

Overall, such results may have been expected as HL dimensions allow people to identify reliable and accurate health information, resolve doubts on their own or with peer support and learn about preventive health services, anticipating and mitigating the occurrence of complications. This perspective is also supported by other authors who associate lower HL with a reduced awareness of AF diagnosis [26], adherence to treatment [20], higher rates of re-hospitalization [27], risk of mortality [27–29] and a decrease in physical and emotional health status [15]. This study [15] also uses the HLQ questionnaire but only evaluated two dimensions, “Understanding health information” and “Engaging with healthcare providers”, and suggests that HL is also associated with health behaviours in cardiovascular patients.

In addition to the above variables, a relationship between age, educational or academic level and social class was also observed with HL scores. Younger patients considered themselves capable of identifying accurate information from different sources, even on their own, remaining up-to-date and knowledgeable about health services. These results are consistent with other studies on patients with cardiovascular health problems, which negatively correlated the level of HL with the age of the participants [30–32]. The powerful influence of ‘educational’ or academic factors on HL, explaining about 60% of the HL level [31], as well as ‘social class’ has been extensively described [14, 33, 34]. These findings coincide with our results where these variables presented significance in all dimensions of HL.

The health outcomes obtained in our study would align well with the WHO’s social determinants model, which proposes the ‘social origin’ of diseases, in turn causing health inequalities. Within this framework, HL has shown its central role as a determinant of health [35]. In fact, low levels of education limit access to better jobs, or nudging persons towards less secure and riskier jobs, with lower incomes and poorer levels of health throughout life [36, 37].

Our research has shown that different health determinants could contribute to inadequate control of anticoagulant therapy. Such determinants would be older age (over 75 years), basic or no education, limited HL (specifically the HL dimensions “Assessment of health information”, “Ability to find good health information” and “Understanding health information sufficiently to know what to do”), low social class, obesity, multi-morbidity and polypharmacy. This finding allows an early identification of a profile of patients, more vulnerable, who would be most at risk of developing complications, having worse health outcomes.

Further, our study is a pioneer in evaluating the nine dimensions that make up HL in patients on VKAs treatment (only one study has been found that evaluated the HL dimensions in this population and it only assesses two dimensions) and we noted the importance of the “social health support” dimension which showed a higher than expected average score. This dimension assesses a person’s social system and measures the following aspects: If I can get access to several people who understand and support me. If when I feel ill, the people around me really understand what I am going through. If I need help, I have plenty of people I can rely on. If I have at least one person who can come to medical appointments with me and if I I have strong support from family or friends.

In our study, this high score could be explained by the environment in which the study was carried out, a mainly rural área where inhabitants would benefit more from social support networks at neighbourhood and family level, less present or weaker in the urban environment, as shown in some studies carried out in Spain on patients with various pathologies [38, 39].

In addition to the novel aspects discussed above, our work establishes a strong relationship between the dimensions of HL and the social determinants that constitute the axes of inequality in health. It highlights the importance of considering HL as a determinant of health because it is a predictor of individual health status and enables people in vulnerable situations to be identified and health inequalities to be addressed more effectively.

For all these reasons, our study shows how HL intervenes in health behaviours and outcomes, modulating therapeutic concordance and the occurrence of complications in cardiovascular patients. Furthermore, taking into account the social determinants that influence HL, it would be appropriate to establish individual therapeutic measures and community interventions that act synergistically in HL to improve the prevention of cardiovascular disease.

### Limitations

The study was conducted only in a rural population setting, using validated questionnaires for Spanish-speaking people. Routine objective variables have been collected and the sample size was calculated to reach a representative sample of the population. Although participation in the study and completion of the questionnaire was offered to all patients, we were forced to stop the collection when the representative sample was reached and this could be interpreted as a selection bias. Furthermore, the study used a bivariate analysis model, so the results should be taken with caution because the statistical significance could not be demonstrated with variables tested in more powerful analyses, such as multivariate models.

## Conclusions

The importance of the social environment in the health and safety outcomes of VKAs treatment is highlighted. People with higher scores on HL dimensions demonstrated better therapeutic control, lower emergency department attendance and fewer hospital admissions, highlighting the importance of intervening on this determinant because of its consequences on the control of patients with heart disease requiring VKAs treatment.

## Abbreviations

AF: Atrial fibrillation
CFI: Comparative Fit Index
DOACs: Direct Oral Anticoagulants
D1: Dimension 1
D2: Dimension 2
D3: Dimension 3
D4: Dimension 4
D5: Dimension 5
D6: Dimension 6
D7: Dimension 7
D8: Dimension 8
D9: Dimension 9
HBP: High Blood Pressure
HL: Health Literacy
HLQ: Health Literacy Questionnaire
INR: International Normalized Ratio
OAC: Oral Anticoagulants
RMSEA: Root Mean Square Error of Approximation
SAHLPA: Short Assessment of Health Literacy in Portuguese-speaking Adults
S-TOFHLA: Test of Functional Health Literacy in Adults
TLI: Tucker Lewis Index
TTR: Time in Therapeutic Range
US: United States
VKAs: Vitamin K antagonists
WHO: World Health Organization
WRMR: Weighted Root Mean Square Residual
